# Effectiveness and safety of different dressings therapy for pressure injuries: A protocol for systematic reviews and network meta-analysis

**DOI:** 10.1097/MD.0000000000023520

**Published:** 2021-01-22

**Authors:** Yitong Cai, Yuying Zhou, Lina Xing, Yingying Kang, Hailing Li, Peng Cheng, Yujuan Wang

**Affiliations:** a Evidence-Based Nursing Center, School of Nursing, Lanzhou University; b Affiliated Hospital of Gansu University of Chinese Medicine; c School of Basic Medical Sciences, Lanzhou University; d Inner Mongolia Medical University; e Department of Orthopedics, Lanzhou University Second Hospital.

**Keywords:** dressing., network meta-analysis, Pressure injuries

## Abstract

**Background:**

Pressure injuries, also known as pressure ulcers, are local skin injuries. Once a pressure injury occurs, clinical treatment is relatively difficult, the treatment cycle is long, and the treatment cost is high, which brings heavy burdens to patients and society. Therefore, look for a reliable pressure injuries treatment method is 1 of the focus of clinical nursing workers.

**Objective:**

At present, there are many kinds of dressings to treat pressure injuries, and there is no uniform conclusion about which dressing is the most effective. Therefore, we systematically evaluate the effects of different dressings on the treatment of pressure injuries.

**Methods:**

We systematically searched the Chinese and English databases: PubMed, Embase, CENTRAL, CINAHL, Web of Science, CNKI, CBM, VIP, Wan Fang. Literature screening, data extraction, and quality evaluation were carried out by 2 researchers, and finally, use R software to carry out network meta-analysis.

**Results:**

This study is ongoing and the results will be submitted to a peer-reviewed journal for publication.

**Ethics and dissemination:**

Ethical approval is not applicable, since this is an overview based on published articles.

**Protocol registration number:**

INPLASY2020100087.

## 1 Introduction

Pressure injuries (PIs), also known as pressure ulcers, is a common and severe complication in clinical nursing work, and is also an important indicator to measure the quality of nursing.^[1,2]^ PIs mainly refers to injuries that occur at bone protuberances and thin areas of fat. It is common in patients who have been bedridden for a long time. It is mainly caused by medical operations or compression by medical devices.^[3]^ Once PIs occurs, it is prone to secondary infection, which will affect the surgical prognosis of patients, lead to the prolonged hospital stay and increased medical costs, which not only increases the burden of treatment and care, but also reduces the quality of life of patients.^[4]^ It not only brings physical and psychological pain to patients, but also is a public economic health problem, which deserves the attention of the majority of medical and health personnel.^[5,6]^ An epidemiological study conducted in Europe showed that the prevalence rate of PIs was 1.8% to 53.2%.^[7–9]^ The medical expenses for treatment and care of PIs accounted for about 3.5% to 5.0% of the national public health expenditure.^[10,11]^ Studies in the United States have shown that pressure ulcers can increase mortality, and deaths from stress injuries to instructors account for 0.4 percent of all deaths in the United States.^[12]^

PIs and its treatment are some of the most challenging clinical problems in hospitals.^[13]^ Therefore, finding a reliable treatment method for pressure ulcers is 1 of the priorities of clinical nursing workers. The current local treatment of PIs mainly uses various wound dressings to promote wound healing, help debride the wound, reduce bacterial load, and prevent further injury. In recent years, there have been more and more studies on dressings to treat PIs. However, at present, there are many kinds of dressings for the treatment of pressure injury, and each has its advantages, and there is no unanimous conclusion.

In the last decade, network meta-analysis (NMA) has been introduced.^[14,15]^ A good NMA of the randomized controlled trial (RCTs) is considered the best quality evidence to provide sufficient information for practice.^[16,17]^ It is also a significant source of critical information for researchers.^[18,19]^ In the same type of research subjects, the NMA can systematically compare several different kinds of interventions for a particular problem, and rank them according to the effect of a specific outcome indicator, to obtain the best intervention plan. Based on this, this NMA evaluated the effects of different dressings in the treatment of pressure injuries, to provide evidence for the clinical selection.

## 2 Methods and analysis

### 2.1 Study registration

This NMA has been registered on the International Platform of Registered Systematic Review and Meta-analysis Protocols (INPLASY). The registration number is INPLASY2020100087, DOI number is 10.37766/inplasy2020.10.0087(https://inplasy.com/inplasy-2020-10-0087/).

### 2.2 Study inclusion and exclusion criteria

#### 2.2.1 Types of studies

Inclusion: RCTs were published in Chinese or English language without restriction on blind methods.

Exclusion:

(1) Non-Chinese and English literature;
(2) Incomplete or missing research data;
(3) Unable to obtain original documents;
(4) Repeated publication of literature;
(5) Editorials
(6) Commentaries.

#### 2.2.2 Types of participants

Patients of any age were described as having PIs. Studies were excluded if the study included other types of wounds (such as chronic wounds and venous leg ulcers) or if the subjects were animals.

#### 2.2.3 Types of interventions

Hydrocolloid dressings, silver dressings, foam dressings, saline gauze, petrolatum gauze, collagen dressings, danghui dressings, honey dressings, and other dressings or conventional treatment.

#### 2.2.4 Types of outcomes measures

Main outcomes:

(1) Effectiveness: Time to complete healing/rate of healing;
(2) Safety: wound infection, bacteria amount, pain during treatment;
(3) Cost.

Additional outcomes: length of hospital stays, the incidence of different types of infection.

### 2.3 Search strategy

#### 2.3.1 Electronic searches

We will search the following English electronic bibliographic databases: PubMed (inception- present), Embase (inception- present), Cochrane Central Register of Controlled Trials (inception- present), CINAHL (inception- present), Web of Science (inception- present), as well as the Chinese databases: China Knowledge Network (inception- present), China Biomedical Literature Database (inception- present), VIP Data(inception- present), Wan Fang Data(inception- present).

#### 2.3.2 Other resources

Furthermore, reference lists of included RCTs and relevant systematic reviews will be searched. There will be no restrictions on publication year.

#### 2.3.3 Search strategies

All databases will be based on the MeSH and text word search will be adjusted according to the specific database. Take PubMed as an example, and the searching strategy is shown in Table 1.

**Table 1:** Searching strategy in PubMed.

#1 “Pressure Ulcer”[Mesh]
#2 pressure ulcer^∗^[Title/Abstract] OR bedsore^∗^[Title/Abstract] OR pressure sore^∗^[Title/Abstract] OR bed sore^∗^[Title/Abstract] OR decubitus ulcer^∗^[Title/Abstract] OR decubital ulcer^∗^[Title/Abstract] OR decubitus ulceration[Title/Abstract] OR decubitus ulcer^∗^[Title/Abstract] OR ulcers decubitus[Title/Abstract])
#3 #1 OR #2
#4 “Randomized Controlled Trials as Topic”[Mesh]
#5 randomized controlled trial [Publication Type] OR random^∗^[Title/Abstract]
#6 #4 OR #5
#7 “Bandages”[Mesh]
#8 “Bandages, Hydrocolloid”[Mesh]
#9 “Occlusive Dressings”[Mesh]
#10 “Honey”[Mesh]
#11 “Hydrogels”[Mesh]
#12 “Alginates”[Mesh]
#13 “Negative-Pressure Wound Therapy”[Mesh
#14“Silver”[Mesh]
#15“Silver Sulfadiazine”[Mesh]
#16 “Collagenases”[Mesh]
#17 Bandage^∗^[Title/Abstract] OR dressing^∗^[Title/Abstract] OR gauze[Title/Abstract] OR tulle[Title/Abstract] OR film^∗^[Title/Abstract] OR bead[Title/Abstract] OR Pad^∗^[Title/Abstract] OR foam^∗^[Title/Abstract] OR hydrocolloid^∗^[Title/Abstract] OR “sodium hyaluronate”[Title/Abstract] OR alginat^∗^[Title/Abstract] OR hydrogel^∗^[Title/Abstract] OR silver^∗^[Title/Abstract] OR honey^∗^[Title/Abstract] OR Foam^∗^[Title/Abstract] OR non-adherent[Title/Abstract] OR “non adherent”[Title/Abstract] OR matrix[Title/Abstract] OR Collagenase^∗^[Title/Abstract]
#18 #7 OR #8 OR #9 OR #10 OR #11 OR #12 OR #13 OR #14 OR #15 OR #16 OR #17
#19 #3 AND #6 AND #18

#### 2.3.4 Literature screening

All search results are imported into ENDNOTE X8 literature management software; Two researchers (YTC, YYZ) were conducted separately, and the relevant literature was screened strictly according to the research purpose and inclusion criteria, and the third researcher (YJW) was requested to judge if there were divergent literature.

#### 2.3.5 Data extraction

After careful reading of the included literature, we will use Microsoft Excel 2013 to create a pre-determined data extraction table to collect relevant information and data, including general data (author, publication date, topic), sample size, intervention measures, and outcome indicators, etc. The data will be extracted independently by 2 reviewers (LNX, YYK). Any differences will be settled through discussions between the 2 reviewers or by the third researcher (HLL).

### 2.4 Study quality assessment

The methodological quality of the final included RCT will be evaluated independently by 2 reviewers (HLL, PC). Any disagreements will be resolved through discussion between the 2 parties or decided by a third reviewer (YJW).

Evaluate the quality of the literature according to the recommended bias risk assessment tool Cochrane 5.1.0. The evaluation contents include: random sequence generation, allocation concealment, blind method of participants, researchers and result evaluators, the integrity of outcome indicators, selective reporting, other source bias, and so on. Each was rated as “high risk of bias,” “unclear,” and “low risk of bias.”

### 2.5 Statistical analysis

#### 2.5.1 Data synthesis

R 3.5.0 Software gemtc package and JAGS 3.4.0 software were used for data analysis, and Stata 15.0 was used to draw the network diagram and funnel diagram. We will calculate the mean differences or standardized mean differences with 95% confidence interval for continuous variable data, and relative risk with 95% confidence intervals for dichotomous variable data. Set the number of pre-iterations to 10,000 and the number of iteration operations to 100,000. The statistical heterogeneity will be examined using the *I*^2^ statistic and *P* value. *I*^2^ was used to judge the size of heterogeneity, *I*^2^ ≤50%, it can be considered that the homogeneity among studies is good; If *I*^2^ > 50%, it is considered that the heterogeneity among studies is large, and multiple regression model is adopted for processing.

#### 2.5.2 Assessment of heterogeneity

The consistency test was judged by the node-splitting model. When *P* < .05, the direct comparison results were inconsistent with the indirect comparison results. If *P* ≥.1, *I*^2^ < 50%, it indicates that there is homogeneity among the studies or the heterogeneity is within the acceptable range, and the fixed effects model is used to merge the calculation of the effect size; on the contrary, it is considered that there is heterogeneity between the studies. Egger method and Begg method were used to assess publication bias.

#### 2.5.3 Subgroup analysis

If the evidence is sufficient, we will conduct a subgroup analysis to determine the difference between different gender, age (Over 60 years old, less than 60 years old, different stages of PIs, courtiers) and so on.

### 2.6 Quality of evidence

Two reviewers (YYC and YJW) will use the Grading of Recommendations Assessment, Development and Evaluation method to assess the quality of evidence of included studies. The evidence levels are classified into 4 levels: high, moderate, low, or very low.

### 2.7 Summary of findings

A “summary of findings” table will be created for the main outcomes, with hydrocolloidal dressing and saline gauze as intervention measures, and the outcome index is mainly the proportion of ulcers completely healed, wound infection, pain during treatment. Finally, we will complete the table after analyzing all the article data, and refer to Table 2 for details

**Table 2:** Summary of findings for the main comparison.

Hydrocolloid compared with Saline gauze						
Setting						
Intervention:Hydrocolloid						
Comparison: Saline gauze						
	**Illustrative comparative risks**^**∗**^** (95% CI)**					
	**Assumed risk**	**Corresponding risk**				
**Outcome**	**Hydrocolloid**	**Saline gauze**	**Relative effect (95% CI)**	**No of participants (studies)**	**Quality of the evidence (GRADE)**	**Comments**
Proportion of ulcers completely healed(Follow up: mean 10 wk)						
wound infection						
pain during treatment						

## 3 Result

**Figure 1:**
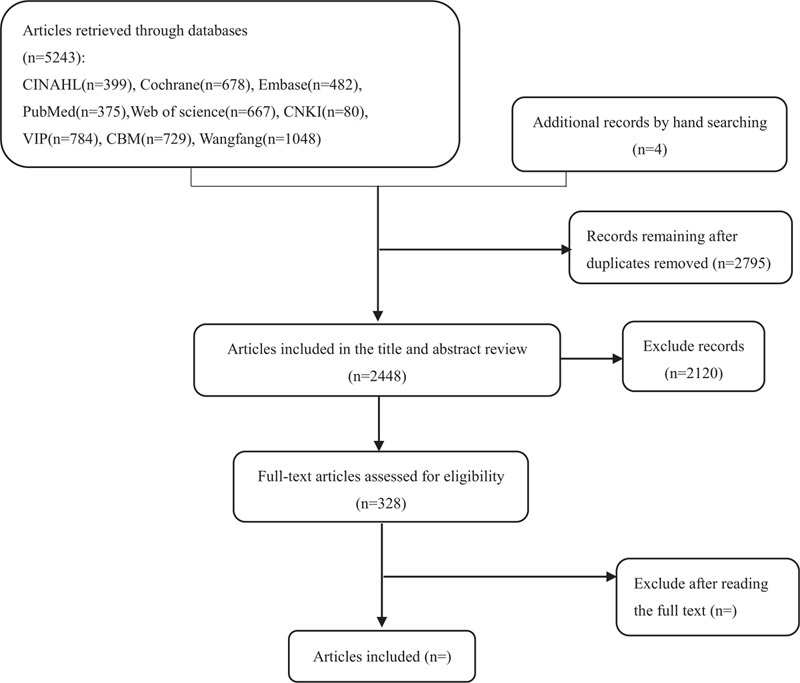
Summary of evidence search and selection.

We identified 5243 records through database searching and 4 records through other sources. The detailed search flowchart is shown in Figure 1.

### 3.1 Characteristics of included studies

We conducted preliminary experiments and included 7 RCTs. Of the 7 RCTs, 2 were from China, 2 were from the United States, 1 was from Iran, and 2 were not mentioned in the article. The total sample size is 299, among the interventions, saline gauze and hydrocolloid dressings are the most, and the subjects are basically pressure ulcer patients with stage 2 and above. For further details, please refer to the characteristics of some of the included studies (Table 3).^[20–26]^

**Table 3:** Characteristics of the included studies.

		interventions		Sample size				
First Author and Year of Publication	Study Location	Treatment	Control	Totol	Treatment	Control	Duration	Stages of pressure ulcers
Hollisaz, 2004^[20]^	Iran	Hydrocolloid	Saline gauze	61	31	30	8 wk	I, II
ZhangL, 2014^[21]^	China	Hydrocolloid	Saline gauze	45	23	22	NR	I, II,III
TangYC, 2016^[22]^	China	Foam	Saline gauze	33	17	16	4 wk	III
Motta, 1999^[23]^	USA	Hydrogel	Hydrocolloid	10	5	5	8 wk	II,III
Avanzi, 2000^[24]^	NR	Adhesive hydrocellular	hydrocolloid	80	NR	NR	3 wk	II,III
Banks, 1994^[25]^	NR	Spyrosorb	Granuflex	40	20	20	NR	II,III
Thomas, 1998^[26]^	USA	Amorphous hydrogel	Saline gauze	30	16	14	10 wk	II,III,IV

## 4 Discussion

Dressings are widely used in wound care, with the aim of protecting the wound and promoting healing. However, there is no consensus on the efficacy, safety and health economy assessment of dressings. So, we did this network meta-analysis to analyze the different dressings and provide a reference for clinical practice.

## Author contributions

**Conceptualization:** Yitong Cai, Yuying Zhou.

**Methodology:** Yitong Cai, Lina Xing, Yingying Kang.

**Software:** Yitong Cai, Hailing Li.

**Writing – original draft:** Yitong Cai, Peng Cheng

**Writing – review & editing:** Yitong Cai, Yujuan Wang.
